# Use of the ADAPTE method to develop a clinical guideline for the improvement of psychoses and schizophrenia care: Example of involvement and participation of patients and family caregivers

**DOI:** 10.1111/hex.13193

**Published:** 2021-02-23

**Authors:** María M. Hurtado, Casta Quemada, José María García‐Herrera, José Miguel Morales‐Asencio

**Affiliations:** ^1^ Mental Health Unit Regional University Hospital Málaga Spain; ^2^ Institute of Biomedical Research of Málaga (IBIMA) Málaga Spain; ^3^ Faculty of Health Sciences University of Málaga Málaga Spain

**Keywords:** ADAPTE methodology, clinical guidelines, patients involvement, psychoses, schizophrenia

## Abstract

**Introduction:**

The aim of this study is to analyse different ways of participation during the development of a clinical guideline to improve the early detection of psychosis and to deploy a comprehensive treatment plan to improve prognosis and social integration.

**Materials and method:**

The clinical guideline was developed using the ADAPTE method with the participation of 40 authors and 80 external reviewers. The process was divided into three major phases: set up, adaptation and finalization. During adaptation and completion, a total of 44 patients and 18 family caregivers were involved.

**Results and conclusions:**

The different roles assumed by the patients and their family caregivers were described, depending on the panel in which they participated, with diverse grades of complexity: a user as author, integration of the results of qualitative research with the participation of local users and family caregivers, 13 users as individual external reviewers and the participation of users and caregiver organizations in the external review. In the guideline, contributions from patients during the qualitative research were included in an innovative way, placing them just behind the recommendations. On the other hand, the results of the family caregivers’ study were included in a specific area of uncertainty. Further, the expressed point of view was considered as the collective demands of users and family caregivers’ organizations in the cost‐benefit analysis made by the organizing committee. There were diverse ways to conduct direct patient participation during the guideline development, ensuring that their individual experiences contributed significantly to the final version.

## 1 INTRODUCTION

Psychosis refers to a set of psychiatric disorders in which an individual's perception, thoughts, mood and behaviour are significantly altered. Schizophrenia is the most common form of psychotic disorder, and because of its complexity and potential severity, it should be a major priority for mental health services.^1^

It is well established that delays in the detection of psychotic disorders usually worsen both the patient's recovery and prognosis.^2^ In addition, management is suboptimal in some cases, with the use of polypharmacy frequently^3^ and insufficient monitoring of the physical health of these people, resulting in excessive mortality with respect to the general population.^4^ These problems may be due to the complexity of the situations associated with psychotic conditions, which can make it difficult for professionals and patients to make decisions. Moreover, suboptimal care for these disorders may also be associated with the lack of implementation of the best knowledge and the existence of unwarranted variations in the provision of effective services or interventions.^5^, ^6^, ^7^, ^8^

Clinical guidelines (CG) are instruments designed to facilitate decision making in complex clinical conditions, as can be considered the case of psychotic disorders.

Nevertheless, consistent results are not obtained in terms of the impact of guidelines implementation on reducing antipsychotic co‐prescription in schizophrenia outpatients.^9^ On the other hand, nurse‐led interventions seem to improve the detection of physical comorbidities.^10^ Eventually, more research is needed to achieve the clinical impact of guidelines on patient outcomes, and alternative ingredients for implementation strategies are necessary as an audit of clinical activities and feedback to doctors.^9^

For example, despite the existence of multiple guidelines for medicine optimization for schizophrenia, the individual's experience of using antipsychotic medication and its implications on adherence and outcomes are scarcely explored, and limited evidence is available.^11^ On the other hand, the participation of patients in the elaboration of CG is an internationally recommended standard by all organizations involved in CG development. Among the reasons that have led to this orientation, it is worth highlighting the provision of more patient‐centred guidelines with recommendations that are more suited to patients’ values and preferences and also facilitate the implementation process.^12^, ^13^ Nevertheless, patient‐reported experience has been poorly developed in people with severe mental illness.^14^

In Spain, in the past 10 years, no CG have focused on psychosis and schizophrenia management. Adaptation methods allow for the development of adapted CG using the best scientific evidence and at a much lower cost than it would have to devise a de novo guide. Alternatively, the integration of patients and their family caregivers throughout the process could provide new approaches that contribute to the improvement of guideline adoption.

The aim of this study is to present the development and elaboration of the Clinical Guideline for the Treatment of Psychosis and Schizophrenia^15^ using the ADAPTE method^16^ and actively promote the participation of stakeholders, including patients and family members.

## 2 METHODS

### 2.1 Study design

A descriptive case study is presented to develop a CG for psychosis and schizophrenia using the ADAPTE method, with the involvement of patients and family caregivers in the process (see Table 1). Multiple methods were used throughout the process for users’ and family caregivers’ participation, such as those intrinsic to the ADAPTE method, together with qualitative methods.

**TABLE 1:** Approaches to users’ engagement in each ADAPTE phase and outcomes in the CG

ADAPTE phase	Approaches to users’ engagement	Outcomes in the CG
Set‐up phase	One user as author in the MP	The participation of users during the entire process ensures respectful and adequate treatment of the user's figure. In addition, there was a person with experience in peer‐support interventions.
Adaptation	Qualitative research with users (n = 30)	The results complement the evidence‐based recommendations, providing adaptation to local users.
		The results influenced the introduction of a new recommendation about work inclusion services.
	Qualitative research with family caregivers (n = 18)	An area of uncertainty specifically dedicated to caregiver care has been introduced, where the main results are summarized.
Finalization	Users as reviewers (n = 13)	Summary and simplification of user experience recommendations.
	Users organizations as reviewers	Detailed analysis of revisions and changes in the wording of the areas of uncertainty.

The ADAPTE method consist of 3 phases, organized through 24 steps to adapt guidelines to a local context considering needs, priorities, legislation, policies and resources.^17^ These three phases are set‐up, adaptation and finalization. In the set‐up phase, an organizing committee (OC) and a multi‐professional panel (MP) are constituted, and the main topic is decided. The second phase of the ADAPTE process includes the determination of specific health questions; the search for existing CG; the assessment of the quality, content, consistency and applicability of existing CG; decision making about adaptation; and the preparation of the first manuscript. Finally, during the third phase, the CG is subjected to an external review, and an update system is included.

### 2.2 Participants and setting

Professionals from mental health clinical practice (psychiatrists, clinical psychologists, mental health nurses), experts on methods for guideline development and implementation, and patients and their families were involved throughout the process.

In the users’ group, a purposive sampling technique with the following criteria was performed: a diagnosis of psychotic disorder made by a psychiatrist or a clinical psychologist according to DMS‐V^18^ criteria, use of public mental health services, and no active symptoms at the beginning of the study that could interfere with participation. Subjects were excluded if they were under the age of 18 or had mental retardation. Thus, the full list of users with a diagnosis of psychotic disorder was obtained from electronic medical records. Once subjects were selected, they were contacted by phone by their mental health nurses to explain the objectives of the study and request their participation. Patients who agreed to participate in the study were afforded sufficient time before the meeting was held to allow them time to read the participant information sheet and ask any questions they considered relevant before signing the informed consent form. The sample size was subject to the principle of saturation of information during data collection and analysis.

For the family caregivers’ group, sampling was aimed towards relatives who have assumed the main caring role for people under mental health services. This selection was carried out by the nursing staff in charge of caring for patients and their families. They telephoned family caregivers following a process similar to the one described above for patients.

The guideline was developed in Málaga (Spain), involving the Regional University Hospital, the University of Málaga and the Andalusian School of Public Health. Health care is provided by the public health‐care system which guarantees universal coverage and free access, including mental health services.

### 2.3 Approaches to engagement

The qualitative phase aimed to foster the involvement of users and family caregivers. A content analysis approach was used, by carrying out two separate groups: users and their family caregivers. This approach was framed in the steps for continuous patient engagement in CG development proposed by Armstrong et al.^12^ Since this framework is not specifically intended for CG adaptation, some steps were not used, such as nominating and prioritizing guideline topics, framing the questions or developing systematic reviews. On the other hand, they were engaged in the rest of the steps proposed by this framework: the creation of an analytical framework (helping refine or expand the scope of the topics or additional factors or situations not covered by the current recommendations), the development of recommendations (according to their preferences and needs, ensuring their readability, providing inputs for potential gaps in the evidence), the dissemination and implementation of recommendations (by consulting barriers and facilitators to implement the CG, involving other users and family organizations, as well as linking their preferences to the recommendations) and the evaluation of methods for impact engagement (by discussing feedback from patient organizations).

Both in the qualitative study carried out with users and in the one carried out with family caregivers, the interviews were semi‐structured and supported by a guide (Annex I). Issues identified in the literature, together with expert consultations and findings from previous studies carried out in our service^19^ were considered for the construction of the interview guide. Finally, the topics included in the guide were the impact of the disorder on the users’ daily life and interpersonal relationships; assessment of their relationships with the professionals who provided care to them (both from primary care and mental health) and the process of care; types of interventions offered by the public health system (including both psychological and pharmacological options) and their perceived impact; and, finally, personal resources used to cope with the disorder. The questions were open‐ended, and the interview was conducted in a flexible style, so that any topic that was not initially proposed was accepted and discussed. The interviews lasted between 90 and 120 minutes and were carried out by neutral interviewers who were experts in group and individual interview techniques and were not associated with the treatment team of the study participants. Likewise, all the interviews included an observer trained for this purpose who was also outside the treatment team. The observer took note of the situation of each participant and the non‐verbal aspects that could help to understand the interactions between the participants. The interviews were conducted in a location that was also different from their usual treatment settings. The interviews were audio‐taped and transcribed verbatim.

Users’ and family caregivers’ contributions were deductively codified according to the recommendations of each uncertainty area. The procedure was similar to that described in previous studies,^20^ which began with a detailed reading of categories and subcategories identified in the patients’ reports and a subsequent process of recognizing commonalities with each recommendation. This linking process was performed by members of the MP and the OC who carried out the qualitative analysis. The initial pairings were distributed to members of the OC to contrast and contextualize these assignments, resolving discrepancies by consensus. On the other hand, the contributions of family caregivers were included in an area of uncertainty specifically aimed at interventions that improve caregivers experience.

To ensure the credibility and validity of the results, the criteria of Guba and Lincoln^21^ were considered: credibility, transferability, consistency and confirmability. To ensure the credibility of the content analysis process, we proceeded to the triangulation of codes and categories. To do this, the coding and classification of the topics was carried out independently by three professionals, consisting of two clinical psychologists with clinical experience as well as in this type of analysis, and a specialist mental health nurse. Transferability was strengthened by the completeness of data collection in each group across multiple potential situations, scenarios and experiences with psychotic disorder. The criteria of data consistency and reproducibility were achieved by a detailed and documented analysis process strategy and the context in which data collection took place. From the point of view of confirmability and reflexivity, before the start of the study, every member of the research team had to perform a reflexivity analysis to identify any preconceived ideas derived from their clinical experience. Moreover, the interviewer was neutral, did not belong to the research team and was highly experienced in conducting qualitative interviews. Qualitative analyses were performed using Atlas Ti 7.^22^

## 3 RESULTS

### 3.1 Phase 1. Set‐up

Following the first phase of the ADAPTE^16^ process, an OC and a MP for the development of the guide were formed. The OC consisted of four psychiatrists, a clinical psychologist, a specialist nurse on mental health, from the Mental Health Service of the Regional University Hospital of Málaga (Spain), a psychologist from the Andalusian School of Public Health, and two academics from the University of Málaga, one of whom was an expert on evidence‐based health care, guideline development and implementation.

Four of these members had experience in developing guidelines using the ADAPTE method in mental health.^23^, ^24^, ^25^

The OC appointed an MP for the development of the guide, which included a large number of professional health‐care staff (16 psychologists, 7 of whom were clinical psychologists; 13 psychiatrists; 6 mental health nurses and 5 primary care physicians). This panel included a woman diagnosed with psychosis who had studied psychology (and thus had technical and scientific training) but had not practised such a profession and participated throughout the entire process. She used to lead peer‐support groups for patients of mental health services for psychosis, and her main contributions focused on the contents in which she was an expert.

### 3.2 Phase 2. Adaptation

Existing CG and other relevant documents were searched. The following organizations and databases were used to search for CG: NICE, SIGN, the National Guidelines Clearinghouse, the Canadian Medical Association Clinical Practice Guidelines, GIN, ICSI and GuíaSalud. As a result of this search, nine guidelines were identified.^26^, ^27^, ^28^, ^29^, ^30^, ^31^, ^32^, ^33^, ^34^ Additional searches were carried out for further information and/or update the evidence concerning the different areas of uncertainty, using PubMed, EMBASE, CINHAL, Cochrane, the Spanish Medical Index, and PsycINFO. Outdated guidelines were discarded, and five of them were selected for the assessment phase^26^, ^27^, ^28^, ^30^, ^34^ (see Table 2). The selected CG were reviewed and evaluated by four independent evaluators using the AGREE II tool^35^ and obtained the results detailed in Table 2.

**TABLE 2:** Summary of the scores obtained in the different guidelines according to the AGREE assessment made by 4 evaluators. CG: Clinical Guidelines. Values expressed in %

	CG 1	CG 2	CG 3	CG 4	CG 5
Quality domain 1. Scope and purpose	88	86	97	97	86
Quality domain 2. Stakeholder involvement	38	54	85	54	46
Quality domain 3. Rigour of development	63	48	95	80	31
Quality domain 4. Clarity of presentation	58	56	75	85	52
Quality domain 5. Applicability	19	31	78	61	22
Quality domain 6. Editorial independence	67	63	71	54	29

Note

CG 1—Royal Australian and New Zealand College of Psychiatrists clinical practice guidelines for the management of schizophrenia and related disorders.^30^

CG 2—Optimal Use Recommendations for Atypical Antipsychotics: Combination and High‐Dose Treatment Strategies in Adolescents and Adults with Schizophrenia.^28^

CG 3—Psychosis and Schizophrenia in adults. The NICE Guideline on Treatment and Management.^27^

CG 4—Management of schizophrenia.^26^

CG 5—Ministry of Health Clinical Practice Guidelines: Schizophrenia.^34^

Based on the results, the NICE guideline was finally selected as the main basis for adaptation according to the AGREE score and the spectrum of intervention areas covered by this guide. However, the SIGN guide was also used as a complement for some areas of uncertainty.

To formulate the initial CG draft, the content of the guide was divided into 16 areas of uncertainty and each area was assigned to a subgroup of participants in the MP, including the users involved in the process. These subgroups aimed to make the Spanish version of the area of uncertainty friendlier, update it and propose possible modifications in the recommendations to the OC, according to its contextualization to the Spanish health‐care system. Two 7‐hour training sessions were carried out to homogenize methodological criteria.

As a result of this process, the descriptions of several areas of uncertainty were modified considering that the result would be more understandable and friendly to Spanish readers. In addition, the OC noted that the NICE guide proposed a strong recommendation on offering cognitive behavioural therapy (CBT) for people at risk of developing psychosis but that it was based primarily on the consensus of experts since the evidence was weak. For this reason, a search of studies was carried out regarding CBT as a therapy for people at risk of developing psychosis from 2013 onwards, resulting in 10 articles. Only randomized controlled trials (RCT) that offered sufficient methodological data were considered, including four articles, three of them on the same RCT, consisting of different follow‐up assessments^36^, ^37^, ^38^ and one independent study.^39^ The GRADE method was applied, and the results of the available evidence remained inconsistent. Furthermore, the OC considered that health services in our country have not been oriented to psychosis risk detection. In fact, within the further external review conducted with professionals, there was no consensus about the feasibility of this recommendation. A benefit‐risk analysis was also conducted. Considering the risk of stigmatization that may develop in our context by attending this type of intervention in the public health system, the weak evidence on its preventive effect and the absence of studies on its cost‐effectiveness in our country, the OC decided to downscale the strength of the aforementioned recommendation.

On the other hand, users’ engagement during this phase consisted of three focus groups and four in‐depth interviews, three of which to people who had already participated in the focus groups. The final number of participants was 30 (15 males and 15 females). Twenty‐four of them had a diagnosis of schizophrenia, four had schizoaffective disorder, one was diagnosed with psychotic disorder and the last with persistent delusional disorder. Likewise, the study devoted to caregivers consisted of two focus groups, with 18 participants (3 males and 15 females, most of whom were the mothers of users).

As a result of this analysis, the users’ and family caregivers’ demands, perceived needs and preferences pointed directly at the recommendations from the CG except the existence of occupational services to promote social inclusiveness for people with psychosis. This proposal emerged from both the MP and users, so the OC discussed the possible consequences of applying this recommendation. The occupational inclusiveness of people diagnosed with serious mental disorders is an on‐going issue in the Spanish context, and the OC decided to accept it as an ‘expert consensus‐based recommendation’.

Afterwards, the OC considered including relevant users’ and family caregivers’ citations along with the CG next to their respective recommendations to connect the evidence with the perceived needs of users and their families. The OC considered that this method of reporting the results from the qualitative phase along with the guideline, just immediately after the recommendation, would produce an additional motivational impact on the clinicians.

For example (see Table 3 for more examples):

**TABLE 3:** Examples of presentation of the results of qualitative studies in the CG

Recommendation: Review antipsychotic medication at least annually, including observed benefits and side‐effects
User testimonials: Most users report that the treatment was prescribed without asking for their preferences and with hardly any information. However, several of them acknowledged having agreed or negotiated the treatment with their therapist, until they found the one that ‘best suited their situation’. *They didn't ask me anything. They told me directly ‘take this’. And they started with a small dose, and then they went up because it didn't work for me, until they got the dose that suits me. But they didn't tell me ‘there is this alternative or the other’. He said to me ‘take this’* (female, 55 years old). *They said ‘you are taking this treatment and it seems to be going well, but it could be even better. Do you want to try another treatment?’ And I said yes because it wasn't too good either* (female, 29 years old). *I have to negotiate a lot. I have been changed thanks to the nurse. I had been undergoing treatment for many years and I was totally down. I had to go to work, drive … and it was a really horrible struggle. They have finally changed me ‐the medicine‐* (female, 50 years old). *When I met this team, they were listening to me, as I was already counting the side effects and what the medication was doing to me. I commented on what was happening to me and they changed it ‐my medication‐* (female, 49 years old).
Recommendation: Treatment with antipsychotic medication should be considered an explicit individual therapeutic trial. Include the following: Discuss and record the side‐effects that the person is most willing to tolerate. Record the indications and expected benefits and risks of oral antipsychotic medication, and the expected time for a change in symptoms and appearance of side‐effects. At the start of treatment give a dose at the lower end of the licenced range and slowly titrate upwards within the dose range given in the drug data sheet. Justify and record reasons for dosages outside the range in the drug data sheet. Record the rationale for continuing, changing or stopping medication, and the effects of such changes. Carry out a trial of the medication at optimum dosage for 4‐6 weeks.
Users testimonial: Most of the users complained of not having received sufficient information about the medicine that was prescribed for them. Someone explicitly commented on how important it would have been to understand what could happen when taking them, especially with regard to weight gain. *I believe that psychiatrists should warn you if there is a risk that (the drug) will make you crave to eat … that they be careful to detect if it makes you want to eat, because it may be the drug* (male, 51 years old). *I was very thin, and I thought ‘I am getting fatter every time’. More and more fat. And I asked ‘Can this medicine make me fat?’ And they told me ‘no, what's up. It doesn't make you fat’. Slimline? And you can´t imagine how much weight I gained* (female, 37 years old).
Recommendation: Offer supported employment programs to people with psychosis or schizophrenia who want to find or return to work
Users testimonials: The participants in the focus groups demanded sheltered or assisted employment programs that would allow them to find or return to work, given their unfavourable work situation because of their diagnoses. *Why isn't there a procedure for the disabled person in that job? Why isn't there an accommodation in their job? Well, if this person does not admit stress, why not set a guideline? This person is disabled for this reason, due to psychotic crisis. The work may increase, but depending on what that person can assume* (female, 26 years old).

Recommendation: ‘Consider offering art therapies to all people with psychosis or schizophrenia, particularly for the alleviation of negative symptoms’.

For this recommendation, several participants reported spending a lot of time doing artistic tasks, obtaining great satisfaction with them as a method for their recovery:

The important thing is to do something that satisfies you … get a drawing and exhibit it … anything that motivates you". (female, 49 years old)

I set goals, such as “for that day, I have to finish this picture.” And the time is coming, and you have it ready. You see yourself as a more normal person… like your sisters or your father when he was working … I don't know … It seems that your life is almost the same as theirs. (male, 40 years old)

### 3.3 Phase 3. Finalization

Lastly, the recommendations for the CG were assessed by an external panel of experts, consisting of 19 psychiatrists, 14 family physicians, 13 nurses (of whom 9 were mental health nurses), 12 psychologists (of whom 9 were clinical psychologists), 2 occupational therapists, 2 social workers, 3 pharmacists and 2 public health technicians (32 males and 35 females). Additionally, 13 patients diagnosed with psychosis participated as external reviewers (8 males and 5 females). All of them had been diagnosed with some type of psychotic disorder, with an average of 22 years since their diagnosis, ranging from those with a first episode within 3 months of diagnosis to those with established schizophrenia diagnoses for 50 years. Their average age was 47 years, ranging between 31 and 66. Their educational levels were distributed as follows: university degree 31%, high school degree 23%, and the rest attended elementary or unknown studies.

This review was carried out individually or in small groups (from two to five people), depending on the needs of the reviewers. A psychologist member of the OC for the CG provided support to them during these sessions, offering them explanations on the task to be performed and, if required, clarifications.

All the reviewers participated voluntarily without receiving any remuneration. The revision process was conducted using a Likert scale from 1 (absolutely disagree) to 5 (absolutely agree).

Of the total 139 recommendations, the external reviewers indicated suggestions or modifications in 47 of them. All the proposals were evaluated and considered by the OC, introducing a total of 22 modifications, 4 of which were related to the content of the recommendation, and 18 were improvements to the wording or clarifications regarding their meaning. The rest of the suggestions were provided without implying any changes.

In general, both the professional and patient reviewers agreed on the complexity of the wording of the adapted recommendations in the ‘User experience of the adult mental health service guide’,^40^ resulting in lengthy recommendations that impeded their understanding. Thus, the wording of this group of recommendations was summarized and simplified. The content modifications focused mainly on the adaptation of some contents to the legislation implemented in Spain, as suggested by two experts in forensic psychiatry.

Most recommendations from the external reviewers were related to the unavailability of some equipment or services in the Spanish context, such as crisis intervention teams or mental health patient advocates. In these cases, the OC considered that it would be preferable to keep these recommendations, because, although they could not be fulfilled at the publication date of the guideline, they were sensitive to many of the perceived demands of users and family caregivers, and could serve as sources of information for patients, caregivers, professionals and decision‐makers on the models of health‐care organizations that have been proven useful. Thus, the CG could guide institutional decision making regarding the future organization of mental health services.

The feature of mental health patient advocate services is quite unknown in our context and raised many concerns among reviewers, specially the non‐professional ones. Nevertheless, given that it has been demanded by patient and family associations for years and will be implemented in the Public Health System of Andalusia in the coming months, the OC decided to keep it as a reference.

The rest of the modifications sought to make the recommendations more understandable or correct wording errors.

Additionally, users and caregiver organizations were also invited to conduct an external review of the CG. As a result, the CG was endorsed by the Association of Relatives of Patients with Schizophrenia in Málaga (AFENES) and the Andalusian Confederation of Relatives and People with Mental Illness (FEAFES).

There were three versions of the *Clinical Guideline for the Treatment of Psychosis and Schizophrenia. Management in primary care and mental health* ^15^ (complete, brief and quick), which can be consulted and downloaded free of charge on the website of the Andalusian Health Service (http://www.juntadeandalucia.es/servicioandaluzdesalud/publicaciones/listadodetalle.asp?idp=715).

## 4 DISCUSSION

This study aimed to present the development and elaboration of the Clinical Guideline for the Treatment of Psychosis and Schizophrenia using the ADAPTE method and actively promoting the engagement of users and family caregivers.

The adaptation of CGs in a context different from the original source is a complex process subject to failure because of cultural, political or health‐care differences. The participation of users and family caregivers in the adaptation process has helped create more tailored versions of the final wording of the recommendations and identify areas that needed further scope. The discussion of recommendations considering the local health system and capacity was guided by the contributions obtained in the qualitative phase. This was a vital step for the successful uptake of the adapted guidelines.^41^

One of the main contributions is the addition of other aspects that were relevant for decision making,^13^ such as the prescription of antipsychotics to prevent psychosis, referrals to primary care, information that users received about their antipsychotic medication prescription, or their side‐effects. Moreover, their values and preferences helped the decision on the strength of some controverted recommendations applied to the local context. Many studies have described the actual disparity between the preferences of patients and those of health‐care professionals,^42^ so the availability of patients’ perspectives in each recommendation is a valuable resource to address this issue. Nevertheless, a long‐term evaluation of the implementation of the CG will be necessary to definitively confirm this impact.

People with psychosis and their family caregivers assumed different roles depending on the panel in which they participated, assuming more extensive or specific tasks. Thus, a user with proper technical and scientific training participated as part of the MP, involved in the scientific literature review and the proposal of changes or new recommendations. Likewise, a group of 30 users and another group of 18 family caregivers participated in qualitative studies that served the local adaptation of the recommendations, and have an influence in the decision of the OC to include a new recommendation about work inclusion services. Finally, two users organizations acted as external reviewers of the CG and 13 users participated as individual external reviewers of the recommendations about user experience.

All participants reported that the experience was highly positive and they were actively involved throughout the full period. This was remarkable since some previous studies on patients’ participation in mental health decision making have reported difficulties among these patients in being perceived as a ‘competent and equal person’.^43^ The supportive attitude among OC members facilitated participation, as was previously reported by similar processes on mental health guideline development.^44^

A commonly reported barrier to users’ involvement in health‐care improvement processes is the difficulty in their implementation.^45^ Similarly, in our study, challenges were found in the coordination of external reviewers because of the extensive number of components. Likewise, during the qualitative phase with patients and their families, it was not easy to reconcile the schedules and availability of many participants, especially during the focus groups. Other user involvement barriers identified in previous studies included the lack of financial support for users and the considerable amount of time required to participate.^46^, ^47^ However, in our case, users with well‐valuated user‐clinician relationships did not show inconvenience in involvement, as reported before.^45^ Nevertheless, this might pose a risk of self‐selection bias. Some studies have reported how poverty and stigma emerge as barriers to users’ involvement,^48^ which cannot be ruled out in our study.

This study has several limitations. First, the adaptation process was carried out for a specific context (the Spanish health‐care system), and some findings would not be generalizable to other contexts. Likewise, the CG was based on the NICE CG, which could be a limitation although it was selected via a formal review and quality assessment process. Some authors have reported an evolution in focus from identifying source guidelines for adaptation to identifying specific recommendations for adaptation.^41^

Users did not participate in the selection of uncertainty as proposed by Armstrong et al^12^ Additionally, we found that users with good user‐clinician relationships participated more than other patients as reported in previous studies.^45^

We did not perform a formal evaluation of satisfaction and the resources employed. Eventually, the final impact of patients’ involvement on the improvement of services for people with psychosis and schizophrenia would require a long‐term sustainability assessment.

## 5 CONCLUSION

The use of adaptation methods has allowed for the development of a CG for people with psychosis and schizophrenia adapted to the Spanish health context, which for more than a decade has not had any resource for decision making in this field of mental health clinical practice. The ADAPTE toolkit is an invaluable resource that guarantees the systematization of the entire process. On the other hand, the comprehensive participation of patients and their families has helped incorporate their values and preferences not only in the writing of the recommendations but also in their contextualization to the local setting and the contribution of new perspectives for decision making.

## CONFLICT OF INTEREST

The authors have no conflicts of interest to declare.

## Data Availability

Data sharing is not applicable to this article as no new data were created or analysed in this study.
